# Structured Exercise During Chemotherapy for Locally Advanced or Metastatic Pancreatic Cancer: A Single‐Arm, Feasibility Trial

**DOI:** 10.1002/cam4.71631

**Published:** 2026-02-16

**Authors:** Anita Borsati, Linda Toniolo, Gloria Adamoli, Christian Ciurnelli, Ilaria Trestini, Lorenzo Belluomini, Daniela Tregnago, Jessica Insolda, Sara Pilotto, Federico Schena, Michele Milella, Alice Avancini

**Affiliations:** ^1^ Department of Neurosciences, Biomedicine and Movement Sciences University of Verona Verona Italy; ^2^ Biomedical, Clinical and Experimental Sciences, Department of Medicine University of Verona Italy; ^3^ Section of Oncology, Department of Engineering for Innovation Medicine (DIMI) University of Verona School of Medicine and Verona University Hospital Trust Verona Italy; ^4^ Dietetic Service, Medical Direction, Azienda Ospedaliera Universitaria Integrata di Verona Verona Italy

**Keywords:** chemotherapy, exercise, pancreatic cancer, physical fitness, quality of life

## Abstract

**Introduction:**

Evidence on structured exercise during systemic treatment for patients with locally advanced or metastatic pancreatic cancer is still limited, and detailed feasibility data in real‐world clinical settings remain scarce. This study aimed to assess the feasibility, safety, and preliminary efficacy of a 12‐week exercise intervention in this population.

**Methods:**

In this prospective single‐arm trial, 20 patients undergoing chemotherapy participated in a supervised exercise program comprising aerobic and resistance training, delivered twice weekly for 12 weeks. Participants could choose between supervised gym‐based or home‐based sessions. Primary outcomes included recruitment, retention, attendance, adherence, tolerance, and safety. Secondary outcomes were changes in physical fitness and patient‐reported outcomes. Descriptive statistics and paired *t*‐tests or Wilcoxon signed‐rank tests were used for analyses. Exploratory subgroup analyses were conducted by tumor stage and exercise delivery mode.

**Results:**

The recruitment rate was 80%, with a 43% dropout rate primarily due to disease progression or treatment‐related toxicities. Median session attendance was 79%, and adherence to prescribed exercise volume reached 77%. Only three non‐serious adverse events were reported. Significant improvements were observed in grip strength (*p* = 0.049), total weekly physical activity (*p* = 0.024), and time spent in moderate‐intensity activity (*p* < 0.001). Emotional (*p* = 0.022) and social functioning (*p* = 0.048) significantly improved, while appetite loss decreased (*p* = 0.010). Subgroup analyses suggested greater benefits in patients with stage IV disease and those in the home‐based group.

**Conclusions:**

Exercise during chemotherapy appears feasible and safe for patients with advanced/metastatic pancreatic cancer, and it may help maintain physical fitness, enhance emotional and social well‐being, and alleviate appetite loss. However, different strategies are required to reduce the dropout rate.

## 1 Introduction

Pancreatic cancer remains one of the most aggressive malignancies, associated with a poor survival, with a five‐year survival rate of approximately 10% [1]. This dismal prognosis is primarily attributable to the aggressive biological nature of pancreatic cancer, since it tends to progress and metastasize rapidly, remaining asymptomatic until an advanced stage of disease [2]. Even when symptoms are present, they are typically nonspecific and often vary depending on the tumor's anatomical location [2]. As a result, approximately 75% of pancreatic malignancies are diagnosed at a locally advanced or metastatic stage [2]. From a therapeutic standpoint, in almost all advanced and metastatic pancreatic cancer cases, systemic treatments, often consisting of chemotherapeutic agents, remain the mainstay of therapy [3].

Pancreatic cancer is associated with substantial adverse effects, even when combined with systemic treatment. Over 85% of patients with advanced or metastatic pancreatic cancer present with significant symptom burden even before the initiation of systemic treatment. Although a transient improvement in symptom severity is often observed during the first 6 months of chemotherapy, symptoms tend to re‐emerge and progressively worsen thereafter [4]. High levels of fatigue, anorexia, pain, and nausea are often reported by the patients [5]. Additionally, impairments in terms of body composition are frequent. In this sense, patients with pancreatic cancer are at high risk of developing sarcopenia and cachexia, with a prevalence of up to 80% [6]. On the psychological corner, anxiety and depression are common in this population, with approximately 13% of patients receiving a first prescription for antidepressants and 20%–30% for anxiolytics or hypnotics within the first 2 years after diagnosis, highlighting the substantial psychological burden associated with pancreatic cancer [7]. From this perspective, supportive strategies aimed at ameliorating symptom burden, overall enhancing patients' quality of life (QoL), are utmost important in the context of advanced/metastatic pancreatic cancer.

Over the years, physical exercise has been demonstrated to be an essential approach for the oncological population. Observational data and a recent interventional study highlight the benefits in terms of survival [8, 9]. At the same time, exercise is a recognized strategy to improve patients' physical fitness, including cardiorespiratory fitness [10, 11], muscle strength, and mass [12], and manage symptoms, such as fatigue [13], anemia [14], and peripheral neuropathy [15], overall enhancing QoL. However, most of the available evidence comes from studies on breast, lung, prostate, and colorectal cancer, mainly conducted in early‐stage settings. Investigations evaluating exercise in advanced/metastatic cancer are still scarce, even more so when focused on pancreatic cancer. To date, two studies have explored the effect of exercise in patients with advanced/pancreatic cancer. One randomized trial has tested the effect of 8‐week supervised strength training on 41 patients with stage IV pancreatic or biliary tract cancers. The intervention was revealed to be feasible, with significant improvements in strength levels, while no changes were observed for QoL [16]. On the other hand, Neuzillet and colleagues randomized 313 patients receiving first‐line chemotherapy to perform a 16‐week combined, home‐based program or usual care. At post‐intervention, there were no significant differences in QoL and survival outcomes between the two groups, despite the low dropout rate reported [17]. However, to be efficacious, several factors, including frequency, duration, intensity, attendance, and adherence to the proposed prescription, should be considered to provide an adequate stimulus to achieve the potential benefits. As mentioned before, advanced/metastatic pancreatic cancer poses unique challenges, especially for the related symptoms, and treatments that may potentially interfere with the exercise intervention. Although recent trials have incorporated mixed‐modality exercise programs in this population, detailed feasibility and implementation data, particularly during active chemotherapy and in real‐world tertiary oncology settings, remain limited. Moreover, previous studies generally adopted a single delivery mode, whereas allowing patients to choose between home‐based or clinic‐based sessions may offer relevant insights into feasibility in this clinically fragile group. At the same time, to our knowledge, no studies have deeply analyzed the feasibility of a combined aerobic and resistance training in this population, thus leaving the literature lacking this critical information. Therefore, the primary aim of this study is to determine the feasibility of a 12‐week aerobic and resistance training program in patients with advanced or metastatic pancreatic cancer undergoing chemotherapy. Moreover, we wanted to explore the effect on physical fitness and QoL, hypothesizing that such intervention would be feasible and effective in this population.

## 2 Methods

### 2.1 Study Design, Participants, and Procedures

This is a prospective, single‐arm study aimed at evaluating the feasibility and preliminary effectiveness of a structured exercise program in patients with advanced/metastatic pancreatic cancer undergoing systemic treatments. Eligible patients were aged ≥ 18 years; had a histologically confirmed diagnosis of locally advanced or metastatic pancreatic cancer; were undergoing systemic treatments according to the current standard of care; had an ECOG performance status of 0 to 2; and signed the written informed consent. Patients were excluded if they had any medical condition that contraindicated exercise. Between 2022 and 2025, eligible patients were screened for eligibility at the Oncology Unit of the University of Verona Hospital Trust. During routine check‐up visits, healthcare providers informed eligible patients about the study and offered them the opportunity to participate. Interested individuals were subsequently contacted by the research staff to arrange an initial visit to the Department of Neuroscience, Biomedicine, and Movement Sciences at the University of Verona. During this meeting, the research staff provided detailed information about the study, obtained informed consent, and conducted the baseline assessments.

The study was conducted following Good Clinical Practice guidelines and adhered to the principles of the Helsinki and Oviedo Declarations. Ethical approval was obtained from the Verona University Ethics Committee for Clinical Trials (Protocol No. 33320). This report follows the CONSORT Statement: extension for randomized pilot and feasibility trials [18].

### 2.2 Exercise Intervention

The exercise program was composed of two weekly sessions of aerobic and resistance training, with all components individually tailored to each patient based on their clinical status and functional assessment. To design the program, different factors were taken into account: (i) medical history and symptoms (i.e., side effects of treatments, concurrent comorbidities, prior surgery procedures, presence of metastases); (ii) the exercise oncology guidelines from different international societies [19, 20, 21]; (iii) baseline assessments, including physical fitness, QoL, and previous exercise experience; (iv) individual preferences and barriers related to exercise delivery (e.g., travel distance, need for supervision). To accommodate personal needs and logistical constraints, patients were given the option to complete all sessions either in a supervised gym setting or independently at home. Participants were not allowed to change the selected exercise delivery mode during the intervention. Home‐based participants received a personalized written exercise program detailing modality, frequency, duration, and target intensity, together with a log diary to track adherence. They also attended three scheduled in‐person meetings with an exercise professional and received weekly follow‐up phone calls to support progression and ensure program fidelity. To equate home‐based and clinic‐based prescriptions, the aerobic component followed the same structure in both settings: participants were instructed to regulate intensity using the 10‐point Borg Rating of Perceived Exertion Scale (target RPE 3–4), allowing walking or preferred aerobic activities at home to match the moderate intensity achieved with treadmill or cycle ergometer during supervised sessions. Duration progressed identically (from 10 to 25 min over 12 weeks), ensuring comparable training volume across delivery modes. Resistance training followed the same prescription as the clinic‐based program, using bodyweight or elastic bands and progressing in sets and repetitions according to the patient's tolerance. Each session consisted of aerobic and resistance training, started with a warm‐up, including 10′ of dynamic stretching, and concluded with a cool‐down (five static stretching exercises). The aerobic training, composed of cardiovascular exercises (e.g., continuous treadmill or cycloergometer for supervised sessions, or walking or preferred aerobic activity for home‐based sessions), was proposed. Duration was initially set at 10 min, with a progressive increase every 2 weeks up to 25 min, while the intensity was moderate, checked using the 10‐point Borg Rating of Perceived Exertion Scale (i.e., 3–4 RPE). Resistance training included five exercises using bodyweight or elastic bands, performed with two sets of 8 repetitions and progressing up to 3 sets of 12 repetitions at moderate to vigorous intensity (i.e., 3–5 RPE). The main aim was to progressively increase the training volume over time, first by increasing the number of repetitions, then the number of sets, and finally by introducing more complex exercises. In addition, one balance exercise was included following resistance training.

### 2.3 Endpoints

#### 2.3.1 Primary Endpoint

The primary study endpoint was the feasibility, assessed by the following: (i) recruitment rate, defined as the proportion of participants referred by the oncology team to the exercise program who provided consent and completed the baseline assessment. During the study period, referral to the program was based on the oncologists' clinical judgment, and no systematic screening log was maintained. Therefore, the total number of potentially eligible patients treated at the center was not available; (ii) completion rate, defined as the ratio between patients enrolled and those who completed post‐intervention assessments; (iii) attendance to aerobic and resistance training, measured as the number of sessions attended out of the total planned. Attendance was further categorized as missed sessions, temporary interruption (≥ 3 consecutive missed sessions), or permanent discontinuation (permanent stop of the program before week 12); (iv) adherence to the prescribed exercise dosage was assessed by calculating, for each participant, the proportion of completed sessions that required modifications. Participants were classified as having dose modifications if ≥ 10% of their sessions required adjustments in exercise intensity or volume or early termination, when sessions were stopped before reaching the intended intensity or duration; (v) tolerance to the program, evaluated by comparing the session RPE to the target RPE values; (vi) safety, monitored by recording exercise‐related adverse events according to the Common Terminology Criteria for Adverse Events (CTCAE), version 5.0 [22]. The study staff monitored the feasibility and incidence of adverse events throughout the study. In line with previous literature, the intervention was deemed feasible if no severe or life‐threatening adverse events occurred and if at least three of the following criteria were met: a recruitment rate above 50%, a loss to follow‐up rate below 20%, median attendance exceeding 80%, median adherence greater than 75%, and tolerance to the target RPE in over 70% of sessions [23, 24, 25].

#### 2.3.2 Secondary Endpoints

Secondary endpoints enclosed a series of physical fitness parameters and patient‐reported outcomes, evaluated at baseline and post‐intervention. Among physical fitness, anthropometric values, including body weight, height, waist, and hip circumferences, were assessed according to the World Health Organization protocols [26], whereas flexibility was evaluated using the standardized chair sit and reach test and back‐scratch test [27]. The handgrip strength test was used to evaluate grip strength [28], and the six‐minute walking test, conducted according to the American Thoracic Society procedures, to estimate cardiorespiratory fitness [29].

Regarding the patient‐reported outcomes, QoL was assessed using the European Organization for Research and Treatment of Cancer Quality of Life Questionnaire Core Module (EORTC QLQ C‐30), which incorporates five functional scales (physical, role, cognitive, emotional, and social), three symptom scales (fatigue, pain, and nausea and vomiting), and a global health and QoL scale. The remaining single items evaluate additional symptoms commonly reported by patients (dyspnea, appetite loss, sleep disturbance, constipation, and diarrhea) [30]. The Godin's Shepard Leisure Time Exercise Questionnaire was employed to record the duration and frequencies of physical activity performed at strenuous, moderate, and mild intensities in the previous week, including both prescribed exercise sessions and any additional activities completed independently by the participants [31].

Sociodemographic information was collected using a dedicated questionnaire, whereas clinical data were extracted by reviewing the medical charts.

### 2.4 Statistical Analysis

Baseline characteristics and feasibility outcomes were summarized using descriptive statistics, including means, standard deviations, medians, interquartile ranges, and percentages. The distribution of secondary outcome variables was tested for normality using the Shapiro–Wilk test. Depending on the data distribution, pre‐ and post‐intervention comparisons were performed using either the Student's *t*‐test or the Wilcoxon signed‐rank test. Statistical analyses were carried out using SigmaStat version 4.0 (Systat Software Inc.). All tests were two‐sided, with a significance threshold set at *p* < 0.05. Due to the exploratory nature of this feasibility study, no formal sample size calculation was performed. Nevertheless, based on existing literature and the number of patients typically treated at the Oncology Unit, a sample of 35 patients was considered appropriate to evaluate feasibility and to explore preliminary effects of the exercise intervention on secondary endpoints.

## 3 Results

**FIGURE 1:**
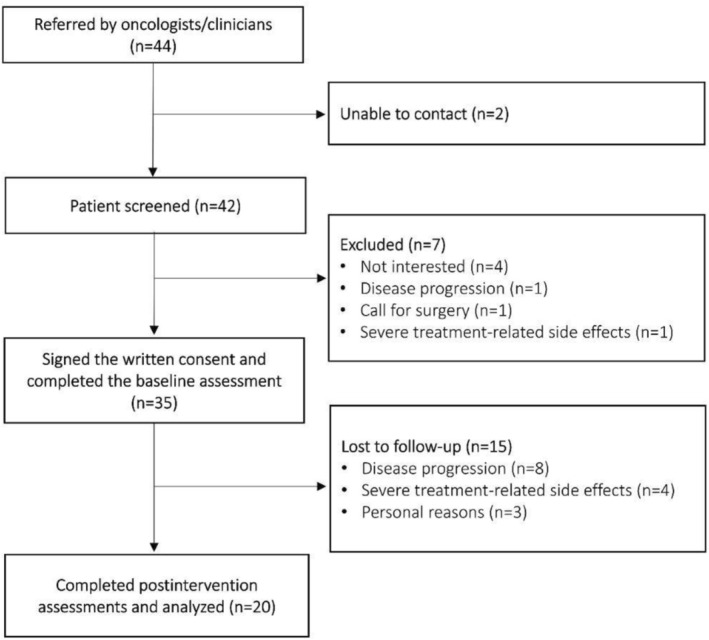
Study flowchart.

Overall, 44 patients were referred to the exercise intervention. Of these, two patients could not be contacted, and 42 were screened for eligibility. Among these, seven patients declined participation due to the lack of interest in participating (*n* = 4), disease progression (*n* = 1), scheduled surgery within the following 3 months (*n* = 1), and a high symptom burden (*n* = 1). A total of 35 patients provided informed consent and underwent the baseline assessments; 23 chose the home‐based, whereas 12 preferred the gym‐based intervention. The study flow diagram is displayed in Figure 1.

### 3.1 Patients' Characteristics

Baseline characteristics of the enrolled patients are presented in Table 1. The majority of participants were male (60%), with a mean age of 60.7 ± 10.0 years. Nearly half of the sample (45.7%) were retired, 34.3% held an undergraduate degree, and 82.8% were married. Adenocarcinoma was the most prevalent histology (91.4%), 74.3% had a metastatic stage of disease, with 51.4% presenting single‐organ metastases. All participants were receiving chemotherapy, and the median time post‐diagnosis was 5 months (IQR: 4.0–8.0 months). About 69% of patients had at least one comorbidity.

**TABLE 1:** Baseline clinical and sociodemographic characteristics of enrolled patients.

Characteristics	Participants (*n* = 35)
Age, mean (SD)	60.7 (10.0)
Male, *n* (%)	21 (60.0)
Female, *n* (%)	14 (40.0)
Education, *n* (%)	
Secondary	10 (28.6)
High school degree	10 (28.6)
Undergraduate degree	12 (34.3)
Postgraduate degree	3 (8.6)
Marital status, *n* (%)	
Married	29 (82.8)
Divorced	3 (8.6)
Single	3 (8.6)
Employment, *n* (%)	
Full‐time employed	12 (34.3)
Part‐time employed	3 (8.6)
Retired	16 (45.7)
Sick leave	4 (11.4)
Family income, *n* (%)	
More than adequate	14 (40.0)
Adequate	16 (45.7)
Barely adequate	5 (14.3)
Stage, *n* (%)	
III	9 (25.7)
IV	26 (74.3)
Tumor histology, *n* (%)	
Adenocarcinoma	32 (91.4)
Neuroendocrine tumor	1 (2.9)
Acinar carcinoma	1 (2.9)
Colloid carcinoma	1 (2.9)
Primary tumor location, *n* (%)	
Body of the pancreas	14 (40.0)
Head of the pancreas	10 (28.6)
Body‐tail of the pancreas	8 (22.8)
Tail of the pancreas	3 (8.6)
Metastatic involvement, *n* (%)	
Single organ	18 (51.4)
Multiorgan	8 (22.9)
Metastases site, *n* (%)	
Liver	20 (57.1)
Lung	6 (17.1)
Peritoneum	3 (8.5)
Lymph nodes	4 (11.4)
Bones	4 (11.4)
Months since diagnosis, median (IQR)	5.0 (4.0–8.0)
Current anticancer treatment status, *n* (%)	
Ongoing	35 (100.0)
Type of treatment, *n* (%)	
Chemotherapy	34 (97.1)
Chemotherapy + immunotherapy	1 (2.9)
Prior surgery	
Yes	10 (28.6)
No	25 (71.4)
Comorbidities	
Diabetes	7 (20.0)
Hypertension	11 (31.4)
Musculoskeletal issues	7 (20.0)
Other^a^	28 (80.0)
Exercise program methods, *n* (%)	
Clinic‐based program	12 (34.3)
Home‐based program	23 (65.7)

^a^ Type of comorbidities: neurological disease (11.4%), endocrine disease (11.4%), gastrointestinal disease (17.1%), other oncological disease (8.5%), urological disease (5.7%), cardiovascular disease (1.0%).

### 3.2 Feasibility and Safety

Feasibility outcomes are summarized in Table 2. The recruitment rate was 80%, and the overall completion rate was 57%, with 15 patients withdrawing from the intervention, mainly due to cancer progression (*n* = 8), severe chemotherapy side effects (*n* = 4), and personal reasons (*n* = 3). No significant differences in baseline assessments were observed between patients who completed the program and those lost to follow‐up (Supporting Information S1). The median attendance rate was 79% (IQR: 70%–89%) and overall, 757 out of 960 prescribed sessions were completed, with aerobic sessions being missed slightly frequently than resistance sessions. In the gym‐based group, attendance was identical for aerobic and resistance components, as both were performed in the same session. In the home‐based group, participants could split their training across different days, which occasionally resulted in aerobic or resistance sessions being missed independently, explaining the slight differences observed between components. The most common reasons for missed sessions were non–health‐related factors (29.6%), such as family obligations, vacations, followed by scheduled chemotherapy infusions on the same day as the planned training session (26.6%) (Supporting Information S1). Eight patients missed three or more consecutive sessions, representing a treatment interruption of 40%. Two patients discontinued the intervention before completing the 3‐month program: one after 7 weeks due to visual impairments caused by disease progression, and the other after 6 weeks due to lack of motivation and limited time availability. Adherence to the prescribed exercise volume was 77% (IQR: 70%–89%). Two patients required exercise dose adjustments in more than 10% of the sessions: one due to severe fatigue, which limited the number of strength exercises performed, and the other due to the need to increase exercise intensity, as the prescribed load was perceived as too low. Overall, all patients reported exercising at the target intensity (i.e., RPE 3–5). Three Grade 1–2 adverse events were registered (dizziness, *n* = 1; nausea, *n* = 1; shoulder pain, *n* = 1) during the exercise sessions.

**TABLE 2:** Feasibility outcomes of the exercise intervention.

Variable	Overall	Aerobic training	Resistance training
Recruitment rate, *n* (%)	35 (80)	—	—
Lost to follow‐up, *n* (%)	15 (43)	—	—
Adherence, median (IQR)	77% (70%–89%)	79% (69%–89%)	73% (65%–92%)
Attendance, median (IQR)	79% (70%–89%)	79% (70%–89%)	79% (70%–93%)
Treatment interruption, *n* (%)	8 (40)	—	—
Permanent discontinuation, *n* (%)	2 (10)	—	—
Missed session, *n* (%)	203 (21)	105 (52)	98 (48)
Dose modification, *n* (%)	2 (10)	—	—
Early session termination, *n* (%)	0 (0)	0 (0)	0 (0)
Tolerability, %	100	100	100

*Note: Lost to follow‐up*, number of patients who did not complete the study; *Adherence*, number of completed planned exercise dosage compared to the total programmed; *Attendance*, number of attended sessions compared to the total prescribed; *Treatment interruption*, number of patients who missed ≥ 3 continuous sessions; *Permanent discontinuation*, number of patients who ended the program before concluding the 12 weeks; *Missed session*, number of sessions not attended by the patients; *Dose modification*, number of patients that required ≥ 10% of sessions dose escalation/reduction; *Early session termination*, number of sessions interrupted before the planned intensity/duration; *Tolerability*, number sessions performed at the planned intensity.

### 3.3 Physical Fitness and Patient‐Reported Outcomes

**FIGURE 2:**
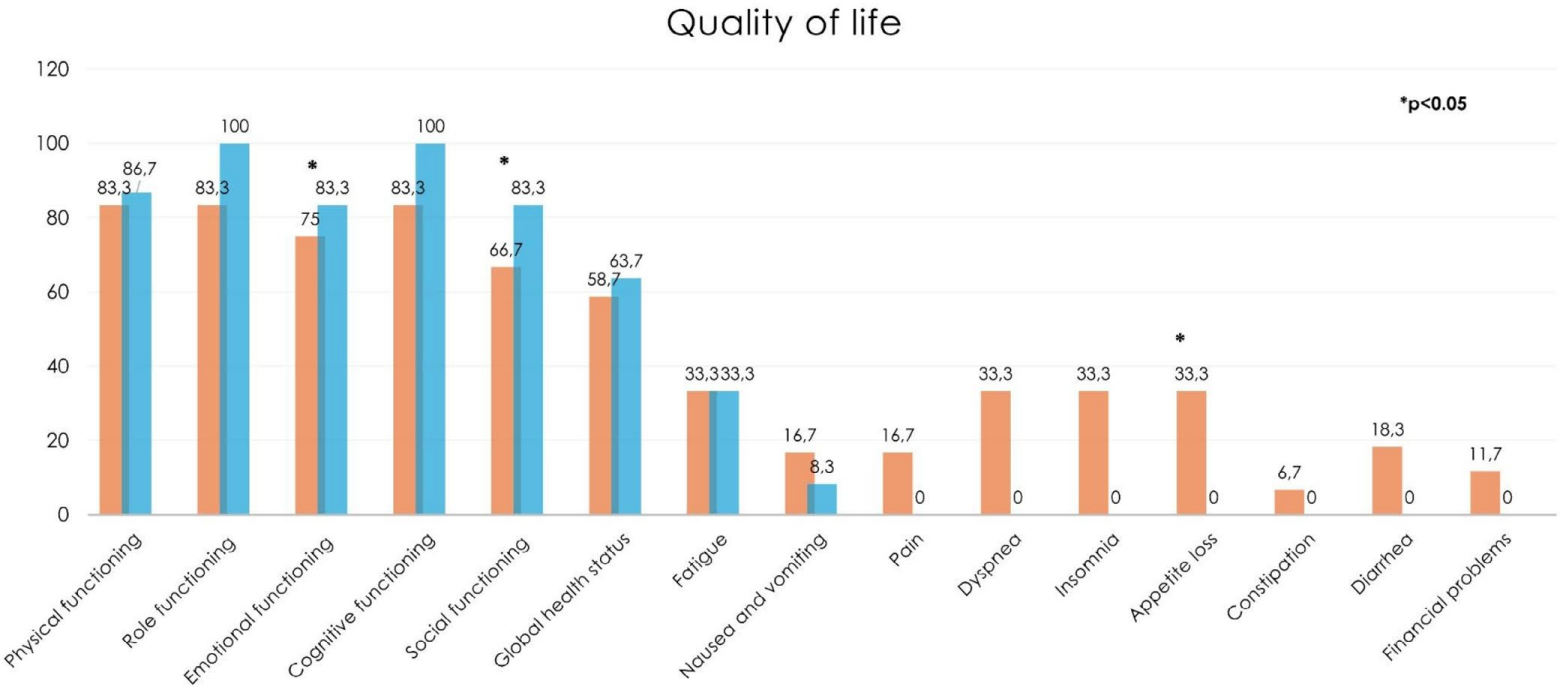
Participants' QoL changes over 12 weeks of physical exercise intervention.

The effects of the 12‐week exercise intervention on secondary endpoints are presented in Table 3. Cardiorespiratory fitness improved without reaching statistical significance (543.3 ± 104.8 vs. 566.2 ± 73.5; *p* = 0.093). A significant gain in grip strength was detected (59.5 ± 20.4 vs. 63.8 ± 17.9 kg; *p* = 0.049). An increase in the total amount of weekly physical activity (190.0 ± 197.8 vs. 326.8 ± 208.9 min; *p* = 0.024) and in the level of moderate intensity physical activity (0.0 [IQR: 0.0–0.0] vs. 105.0 [IQR: 20.0–270.0] points; *p* < 0.001) was reported. Changes in QoL domains from baseline to post‐intervention are displayed in Figure 2. Statistically significant improvements were observed in emotional functioning (75.0 [IQR: 62.5–83.3] vs. 83.3 [IQR: 75.0–91.7] points; *p* = 0.022) and social functioning (66.7 [IQR: 50.0–83.3] vs. 83.3 [IQR: 66.7–100.0] points; *p* = 0.048). Moreover, appetite loss significantly decreased (33.3 [IQR: 0.0–66.7] vs. 0.0 [IQR: 0.0–33.3] points; *p* = 0.010). No other differences were observed.

**TABLE 3:** Participants' physical fitness and physical activity changes over 12 weeks of physical exercise intervention.

Variables	Baseline, mean (SD)	Postintervention, mean (SD)	*p*‐value
Anthropometric measures			
Body weight (kg)	68.41 (12.46)	69.38 (12.43)	0.175
Body mass index (kg/m^2^)	23.95 (4.03)	24.26 (3.76)	0.205
Waist (cm)	86.11 (13.65)	85.53 (12.99)	0.635
Hip (cm)	96.15 (6.38)	96.41 (6.45)	0.835
Waist‐hip ratio (cm)	0.89 (0.10)	0.89 (0.11)	0.542
Chair sit and reach (cm)			
Right leg	−5.20 (13.08)	−4.67 (14.42)	0.773
Left leg^a^	−6.00 (−17.00; 5.50)	−6.75 (−20.25; 5.75)	0.956
Back scratch (cm)			
Right arm	−6.79 (14.01)	−5.07 (12.74)	0.132
Left arm	−11.15 (14.18)	−10.30 (14.05)	0.400
Handgrip (kg)	59.50 (20.43)	63.82 (17.89)	0.049
Six‐minute walking test (m)^b^	543.27 (104.81)	566.22 (73.46)	0.093
Physical activity level (min/week)			
Vigorous	0.00 (0.00–0.00)	0.00 (0.00–0.00)	0.250
Moderate	0.00 (0.00–00.00)	105.00 (20.00–270.00)	< 0.001
Light	65.00 (0.00–210.00)	65.00 (5.00–127.50)	0.940
Total	190.00 (197.88)	326.84 (208.95)	0.024

^a^ Data presented as median and interquartile range.

^b^ Mean excludes two patients who did not perform the test due to severe fatigue and walking impairment related to multiple sclerosis.

### 3.4 Subgroup Analysis According to Exercise Delivery Modality

An exploratory analysis according to the exercise delivery method is reported in the Supporting Information S1. At baseline, the home‐based and clinic‐based groups were balanced in terms of sociodemographic and clinical characteristics. Dropouts occurred more frequently in the home‐based group (48% vs. 33%), whereas patients in the gym‐based program showed a slightly lower attendance compared to those in the home‐based group (77% [IQR: 70%–84%] vs. 79% [IQR: 72%–94%]).

A significant between‐group difference was found for hip circumference (*p* = 0.026), increasing in the home‐based group (*p* = 0.049) and decreasing in the gym‐based program (*p* = 0.216). No other significant differences in physical fitness outcomes were detected. In terms of QoL, appetite loss significantly decreased in the home‐based group (*p* = 0.031), although these patients also reported a significant increase in constipation (*p* = 0.031). Finally, the global health status significantly improved in the home‐based program (*p* = 0.027) with no changes observed in the gym‐based group, resulting in a significant between‐group difference (*p* = 0.039). This was accompanied by a marked increase in moderate‐intensity physical activity particularly in participants assigned to the home‐based program.

### 3.5 Subgroup Analysis According to Cancer Stage

At baseline, patients with stage III and stage IV pancreatic cancer were comparable in terms of sociodemographic and clinical variables (Supporting Information S1). Most dropouts occurred among patients with metastatic disease (87% vs. 13%). The overall attendance rate was slightly lower in stage IV patients compared to stage III (75% [IQR: 71%–92%] vs. 79% [IQR: 73%–85%]), though adherence rates were similar between the groups.

Patients with stage IV disease showed a significant increase in grip strength (*p* = 0.039) and in the six‐minute walking test (*p* = 0.031), while no significant changes were observed among patients with stage III. Conversely, only in the stage III group, an improvement in role functioning (*p* = 0.031), global health status (*p* = 0.012), and a reduction in nausea and vomiting (*p* = 0.038) were observed. Emotional functioning improved significantly in patients with stage IV (*p* = 0.037), resulting in a significant between‐group difference (*p* = 0.041). A significant difference between groups was also observed for pain (*p* = 0.010), which decreased in stage III patients but remained stable in those with metastatic disease.

## 4 Discussion

Trials exploring the feasibility and efficacy of exercise interventions in patients with locally advanced or metastatic pancreatic cancer undergoing treatments are still scarce.

Our intervention met 5 of 6 predefined feasibility criteria. Except for the completion rate, which, at 57%, fell below our target threshold of 80%, we observed high recruitment and attendance rates, good adherence to the prescribed exercise volume, optimal tolerability, and a favorable safety profile, with only three non‐serious adverse events reported. Our recruitment rate of 80% was comparable to prior exercise trials [17, 32, 33]. Similar to the study of De Lazzari et al., our intervention was conducted at a national referral center offering high standards of care [16]. Many patients, despite residing in other regions of the country, were willing to participate, likely facilitated by the flexibility to choose between a home‐based and gym‐based program. This flexibility may have positively influenced not only recruitment but also attendance at exercise sessions. Patients with this diagnosis are known to be highly burdened not only by symptoms and treatment toxicities but also by frequent hospital appointments and clinical routine exams. Offering two modes of delivery likely helped reduce logistical and physical barriers to participation, with no notable differences in feasibility or safety outcomes between the two groups. In exploratory subgroup analyses, some differences emerged according to exercise delivery mode. Home‐based participants benefited from greater logistical flexibility, as aerobic and resistance components could be performed on separate days and adapted to daily symptoms and treatment‐related fluctuations. This flexibility may have facilitated engagement and adherence, particularly in periods of increased symptom burden. In contrast, gym‐based participants were required to complete both components within a single supervised session, which may have limited tolerance to higher aerobic workloads on days of reduced physical capacity. Although these findings should be interpreted with caution, they support the notion that flexible, symptom‐adapted delivery models may help reduce participation barriers in this highly symptomatic population, particularly among patients with more advanced disease. Additionally, our study showed high adherence to the prescribed aerobic and resistance training volumes, with only one patient requiring a reduction due to severe fatigue. Compared with the trial by Luo et al., which adopted similar feasibility metrics, our intervention achieved higher attendance (79% vs. 55%), lower permanent discontinuation (10% vs. 36%), fewer dose adjustments (10% vs. 74%), and no early terminations (0% vs. 10.5%) [34]. We attribute these differences to the flexibility of delivery and the individualized, symptom‐responsive approach adopted. While Luo's prescription was closer to general cancer exercise guidelines, it may have been overly demanding for a population undergoing active chemotherapy. For such clinically fragile patients, achieving recommended exercise volumes should be pursued gradually through a progressive, symptom‐adapted model, accounting for chemotherapy timing and daily symptom fluctuations. This is further supported by the lower number of adverse events observed in our study (3 vs. 9). Despite the high adherence among those who completed the program, the overall dropout rate in our study was relatively high, mainly due to disease progression and the associated clinical deterioration, a pattern consistent with previous research [16, 34]. Indeed, our dropout rate was similar to that reported by Luo et al. in another 12‐week program. By contrast, De Lazzari et al. observed a lower dropout rate (24%) in a shorter, 8‐week program of thrice‐weekly resistance training in patients with stage IV pancreatic or biliary tract cancer, with most withdrawals similarly attributable to disease progression, death, or hospitalization. Collectively, these findings point to a key consideration. A 12‐week intervention may be too long for many patients with advanced or metastatic pancreatic cancer, particularly when recruitment occurs several months after diagnosis, as in our sample (median time since diagnosis of 5 months). In this sense, given the poor prognosis and biological aggressiveness of pancreatic cancer, shorter programs and careful modulation of exercise intensity and frequency should be further explored to maximize benefits while minimizing the risk of attrition.

Regarding anthropometric parameters, although not statistically significant, we observed a slight increase in body weight and BMI, accompanied by a reduction in waist circumference after the exercise intervention. In the absence of body composition data, the exact nature of these changes is difficult to interpret. In pancreatic cancer, where up to 40% of patients lose > 10% of their body weight within 6 months of diagnosis, even weight maintenance is clinically meaningful, as weight loss, often accompanied by muscle wasting and sarcopenia, can impair chemotherapy tolerance, QoL, and survival [35, 36, 37]. In our study, grip strength, a prognostic marker, improved significantly, suggesting preservation of muscle function [38]. While most physical outcomes did not show significant gains, we observed general stability or improvement across several domains. In the absence of a control group, the exact contribution of exercise to these findings remains uncertain. However, given the aggressive nature of pancreatic cancer, it is plausible that, without intervention, these parameters would have declined over the treatment trajectory. This supports the hypothesis that exercise may help prevent deterioration in physical status and functional capacity, reinforcing its role as a supportive strategy in this patient population.

For QoL, most domains remained stable or slightly deteriorated, leading to similar considerations for the lack of a control group as mentioned previously. However, improvements were observed for the domains of emotional and social functioning. Exercise is known to exert anti‐inflammatory effects, modulate neurotransmitter systems, and improve neuroendocrine regulation, significantly improving anxiety and depression levels. This may have contributed to enhancing the emotional well‐being of patients, and it is of utmost importance, especially considering that pancreatic cancer shows one of the highest rates of depression among all malignancies [39]. On the social front, patients often experience social isolation, which may stem from physical limitations, psychological distress, or the disruption of daily life caused by frequent hospital visits and treatments. Social functioning is particularly relevant in this population, as the disease and its management can impact relationships with partners and children, reduce the ability to work, and limit participation in social activities. In this context, our exercise intervention may have contributed to improvements in both physical capacity and psychological well‐being, enabling patients to re‐engage more actively in social interactions. While the exact mechanisms underlying this outcome remain speculative, these findings suggest that exercise could play a role in mitigating the social consequences of cancer and its treatment. Lastly, a significant reduction in loss of appetite was reported by patients at post‐intervention assessment. Appetite loss is a common symptom in patients with advanced or metastatic pancreatic cancer and is associated with an increased risk of malnutrition, anorexia, or cachexia, which can negatively affect survival [40]. From a biological perspective, appetite regulation is governed by a complex interplay between central and peripheral signals [41]. In pancreatic cancer, multiple tumor‐ and treatment‐related factors, such as inflammatory cytokines, contribute to the disruption of central appetite regulation, activating anorexigenic pathways (e.g., POMC/CART) and suppressing orexigenic signaling (e.g., NPY/AgRP), thus reducing food intake [41, 42]. Exercise has shown promising results in the modulation of appetite‐related hormones, reduction of inflammation via the release of anti‐inflammatory myokines, and supporting the maintenance of skeletal muscle mass and energy balance [43]. Finally, exercise can improve mood, motivation, and QoL, potentially restoring interest in food by reactivating reward‐related neural circuits [42]. Exploratory analyses also suggested some differences in outcomes according to cancer stage. Patients with stage IV disease generally showed improvements in grip strength and cardiorespiratory fitness, whereas those with stage III disease reported gains in selected QoL domains, including role functioning and global health status, along with reductions in symptoms such as pain, nausea, and vomiting. These apparent differences are difficult to interpret given the small sample size and single‐arm design, and no causal inferences can be drawn. However, they may reflect the distinct clinical trajectories and symptom burden associated with disease stage. Patients with metastatic disease are often more functionally impaired, which may limit the magnitude of perceived QoL improvements, except for emotional functioning, while patients with locally advanced disease may tolerate higher exercise volumes and experience greater symptom relief.

This study adds to the limited body of evidence on exercise interventions in patients with advanced or metastatic pancreatic cancer and provides a broader understanding of the potential impact of exercise in this setting. Unlike most previous studies that used a single mode of delivery, our flexible home‐ or clinic‐based approach allowed us to capture feasibility under real‐world patient preferences, a relevant aspect in this highly symptomatic and understudied population. The absence of a control group is probably the main weakness of our study, precluding causal inferences regarding the effects of the intervention, and the small sample size and single‐center design further limit generalizability and statistical power. In addition, the recruitment rate was calculated based on the number of patients referred to the program rather than all potentially eligible patients treated at the center, as systematic eligibility tracking was not implemented. This may have resulted in a more selected sample, and future studies should incorporate structured referral logs to more accurately capture the full pool of eligible patients.

In conclusion, this study demonstrates that a flexible, symptom‐adapted exercise program is feasible and safe for patients with advanced pancreatic cancer undergoing systemic treatment. The findings also suggest that exercise may help maintain physical fitness, improve emotional and social functioning, and mitigate appetite loss. Future randomized controlled trials with larger samples and shorter, stage‐appropriate interventions are warranted to confirm these findings and better define the role of exercise in the supportive care of patients with pancreatic cancer.

## Author Contributions

**Anita Borsati:** conceptualization (equal), data curation (equal), formal analysis (equal), investigation (equal), methodology (equal), resources (equal), visualization (equal), writing – original draft (equal), writing – review and editing (equal). **Linda Toniolo:** data curation (equal), formal analysis (equal), investigation (equal), resources (equal), visualization (equal), writing – review and editing (equal). **Gloria Adamoli:** data curation (equal), formal analysis (equal), investigation (equal), methodology (equal), validation (equal), visualization (equal), writing – review and editing (equal). **Christian Ciurnelli:** investigation (equal), visualization (equal), writing – review and editing (equal). **Ilaria Trestini:** resources (equal), visualization (equal), writing – review and editing (equal). **Lorenzo Belluomini:** resources (equal), writing – review and editing (equal). **Daniela Tregnago:** resources (equal), visualization (equal), writing – review and editing (equal). **Jessica Insolda:** resources (equal), visualization (equal), writing – review and editing (equal). **Sara Pilotto:** conceptualization (equal), data curation (equal), project administration (equal), supervision (equal), visualization (equal), writing – review and editing (equal). **Federico Schena:** resources (equal), supervision (equal), visualization (equal), writing – review and editing (equal). **Michele Milella:** conceptualization (equal), data curation (equal), project administration (equal), supervision (equal), visualization (equal), writing – review and editing (equal). **Alice Avancini:** conceptualization (equal), data curation (equal), formal analysis (equal), investigation (equal), methodology (equal), project administration (equal), resources (equal), software (equal), supervision (equal), validation (equal), visualization (equal), writing – original draft (equal), writing – review and editing (equal).

## Funding

The authors declare that no funds, grants, or other support were received during the preparation of this manuscript.

## Ethics Statement

This study was performed in line with the principles of the Declaration of Helsinki and Oviedo. The project was reviewed and approved by the Ethics Committee for Clinical Trials of the University of Verona (Prot. N. 33,320).

## Consent

Written informed consent and consent to publish were obtained from all study participants.

## Conflicts of Interest

S.P. reports personal fees from AstraZeneca, Eli‐Lilly, Novartis, AMGEN, BMS, Boehringer Ingelheim, Merck & Co., and Roche and grants from AstraZeneca and BMS outside of the submitted work. M.M. reports personal fees from Pfizer, MSD, AstraZeneca, EUSA Pharma, Boehringer Ingelheim, and Ipsen and grants from Roche and BM outside of the submitted work. All remaining authors have no conflicts of interest to disclose.

## Supporting information


**Table S1:** Baseline characteristics of completers and dropouts.
**Table S2:** Reason for the missed session of the entire cohort.
**Table S3:** Baseline patient characteristics by exercise program modality.
**Table S4:** Feasibility outcomes by exercise program modality.
**Table S5:** Reasons for missed sessions by exercise program modality.
**Table S6:** Changes in physical fitness by exercise program modality.
**Table S7:** Changes in quality of life and physical activity levels by exercise program modality.
**Table S8:** Baseline patient characteristics by tumor stage.
**Table S9:** Feasibility outcomes stratified by tumor stage.
**Table S10:** Reasons for missed sessions by tumor stage.
**Table S11:** Changes in physical fitness by tumor stage.
**Table S12:** Changes in quality of life and physical activity levels by tumor stage.

## Data Availability

The data supporting the findings of this study are available within the article and/or its supporting materials.
