# Supplementary material for: Structured Exercise During Chemotherapy for Locally Advanced or Metastatic Pancreatic Cancer: A Single‐Arm, Feasibility Trial
Source: Cancer Med. 2026 Feb 16;15(2):e71631. doi: 10.1002/cam4.71631 (PMC12910164; doi:10.1002/cam4.71631)
Supplement: Supplementary file 1 — Table S1: Baseline characteristics of completers and dropouts. Table S2: Reason for the missed session of the entire cohort. Table S3: Baseline patient characteristics by exercise program modality. Table S4: Feasibility outcomes by exercise program modality. Table S5: Reasons for missed sessions by exercise program modality. Table S6: Changes in physical fitness by exercise program modality. Table S7: Changes in quality of life and physical activity levels by exercise program modality. Table S8: Baseline patient characteristics by tumor stage. Table S9: Feasibility outcomes stratified by tumor stage. Table S10: Reasons for missed sessions by tumor stage. Table S11: Changes in physical fitness by tumor stage. Table S12: Changes in quality of life and physical activity levels by tumor stage. [file CAM4-15-e71631-s001.docx]

**Supplementary materials**

**Table S1.** Baseline characteristics of completers and dropouts

**Table S2**. Reason for missed session of the entire cohort

**Table S3.** Baseline patient characteristics by exercise program modality

**Table S4.** Feasibility outcomes by exercise program modality

**Table S5.** Reasons for missed sessions by exercise program modality

**Table S6.** Changes in physical fitness by exercise program modality

**Table S7**. Changes in quality of life and physical activity levels by exercise program modality

**Table S8.** Baseline patient characteristics by tumor stage

**Table S9.** Feasibility outcomes stratified by tumor stage

**Table S10.** Reasons for missed sessions by tumor stage

**Table S11.** Changes in physical fitness by tumor stage

**Table S12.** Changes in quality of life and physical activity levels by tumor stage

**Table S1.** Baseline characteristics of completers and dropouts

| **Characteristics** | **Completers (n=20)** | **Drop-out (n=15)** | **p-value** |
| --- | --- | --- | --- |
| Age, mean (SD) | 60.8 (9.7) | 60.7 (10.8) | 0.985 |
| Male, n (%) | 12 (60.0) | 9 (60.0) | 1.000 |
| Female, n (%) | 8 (40.0) | 6 (40.0) |  |
| Education, n (%) | | | |
| Secondary | 3 (15.0) | 7 (46.7) | 0.237 |
| High school degree | 7 (35.0) | 3 (20.0) |  |
| Undergraduate degree | 8 (40.0) | 4 (26.7) |  |
| Postgraduate degree | 2 (10.0) | 1 (6.7) |  |
| Marital status, n (%) | | | |
| Married | 17 (85.0) | 12 (80.0) | 0.314 |
| Divorced | 2 (10.0) | 1 (6.7) |  |
| Single | 1 (5.0) | 2 (13.3) |  |
| Employment, n (%) |  |  |  |
| Full-time employed | 7 (35.0) | 5 (33.3) | 0.281 |
| Part-time employed | 2 (10.0) | 1 (6.7) |  |
| Retired | 7 (35.0) | 9 (60.0) |  |
| Sick leave | 4 (20.0) | 0 (0.0) |  |
| Family income, n (%) |  |  |  |
| More than adequate | 10 (50.0) | 4 (26.7) | 0.350 |
| Adequate | 8 (40.0) | 8 (53.3) |  |
| Barely Adequate | 2 (10.0) | 3 (20.0) |  |
| Stage, n (%) | | | |
| III | 7 (35.0) | 2 (86.7) | 0.147 |
| IV | 13 (65.0) | 13 (86.7) |  |
| Tumor histology, n (%) | | | |
| Adenocarcinoma | 17 (85.0) | 15 (100) | 0.482 |
| Neuroendocrine tumor | 1 (5.0) | 0 (0.0) |  |
| Acinar cell carcinoma | 1 (5.0) | 0 (0.0) |  |
| Colloid carcinoma | 1 (5.0) | 0 (0.0) |  |
| Primary tumor location, n (%) | | | |
| Body of the pancreas | 8 (40.0) | 6 (40.0) | 0.252 |
| Head of the pancreas | 8 (40.0) | 2 (13.3) |  |
| Body-tail of the pancreas | 3 (15.0) | 4 (26.7) |  |
| Tail of the pancreas | 1 (5.0) | 2 (13.3) |  |
| Metastatic involvement, n (%) | | | |
| Single organ | 8 (40.0) | 11 (73.3) | 0.118 |
| Multiorgan | 6 (30.0) | 2 (13.3) |  |
| Metastases site, n (%) | | | |
| Liver | 11 (55.0) | 9 (60.0) | 0.287 |
| Lung | 5 (25.0) | 1 (6.7) |  |
| Peritoneum | 0 (0.0) | 3 (20.0) |  |
| Lymph nodes | 3 (15.0) | 1 (6.7) |  |
| Bone | 3 (15.0) | 1 (6.7) |  |
| Months since diagnosis, median (IQR) | 7.0 (4.0; 9.3) | 4.0 (3.5; 5.0) | 0.860 |
| Current anticancer treatment status, n (%) |  |  |  |
| Ongoing | 20 (100) | 15 (100) | 0.184 |
| Type of treatment, n (%) | | | |
| Chemotherapy | 16 (94.1) | 15 (100) | 0.390 |
| Chemotherapy + immunotherapy | 1 (5.9) | 0 (0.0) |  |
| Prior surgery, n (%) |  |  |  |
| Yes | 7 (35.0) | 3 (20.0) | 0.331 |
| No | 13 (65.0) | 12 (80.0) |  |
| Comorbidities, n (%) |  |  |  |
| Diabetes | 3 (15.0) | 4 (26.7) | 0.912 |
| Hypertension | 6 (30.0) | 5 (33.3) |  |
| Metabolic syndrome | 2 (10.0) | 1 (6.7) |  |
| Other | 15 (75.0)^a^ | 13 (86.7)^b^ |  |
| Exercise program methods, n (%) | | | |
| Gym-based program | 8 (40.0) | 4 (2.7) | 0.411 |
| Home-based program | 12 (60.0) | 11 (7.3) |  |
| Anthropometric measures, mean (SD) |  |  |  |
| Body weight (kg) | 68.4 (12.5) | 65.1 (14.4) | 0.471 |
| Body mass index (kg/m^2^) | 24.0 (4.0) | 23.2 (3.6) | 0.573 |
| Waist (cm) | 86.1 (13.7) | 83.9 (14.6) | 0.643 |
| Hip (cm) | 96.1 (6.4) | 92.3 (7.6) | 0.115 |
| Waist-hip ratio (cm) | 0.9 (0.1) | 0.9 (0.1) | 0.749 |
| Chair sit and reach (cm), mean (SD) |  |  |  |
| Right leg | -5.2 (13.1) | -2.6 (11.2) | 0.542 |
| Left leg | -6.2 (3.4) | -1.4 (10.5) | 0.255 |
| Back scratch (cm), mean (SD) |  |  |  |
| Right arm | -6.8 (14.0) | -5.6 (11.2) | 0.794 |
| Left arm | -11.1 (14.2) | -12.2 (10.1) | 0.809 |
| Handgrip (kg) | 59.5 (20.4) | 64.0 (17.4) | 0.497 |
| 6-min walking test (m) | 548.4 (104.3)^c^ | 470.2 (122.1) | 0.053 |
| EORTC QLQ-C30, mean (SD) |  |  |  |
| Physical functioning | 83.6 (13.4) | 77.8 (17.4) | 0.272 |
| Role functioning | 75.8 (17.5) | 63.3 (28.3) | 0.117 |
| Emotional functioning | 70.8 (20.8) | 68.9 (26.6) | 0.810 |
| Cognitive functioning | 85.4 (16.6) | 76.7 (21.6) | 0.185 |
| Social functioning | 68.3 (20.9) | 61.1 (31.9) | 0.424 |
| Fatigue | 35.3 (19.9) | 39.3 (26.5) | 0.614 |
| Nausea/vomiting | 15.8 (14.8) | 8.9 (13.9) | 0.168 |
| Pain | 16.7 (20.2) | 10.0 (13.8) | 0.281 |
| Dyspnea | 20.0 (19.9) | 22.2 (30.0) | 0.794 |
| Insomnia | 20.0 (19.9) | 15.6 (27.8) | 0.281 |
| Appetite loss | 36.7 (35.7) | 28.9 (37.5) | 0.489 |
| Constipation | 6.7 (13.7) | 20.0 (27.6) | 0.135 |
| Diarrhea | 18.3 (25.3) | 2.2 (8.6) | 0.026 |
| Financial problems | 11.7 (16.3) | 15.5 (27.8) | 0.984 |
| Global health status | 58.8 (19.2) | 60.0 (23.2) | 0.863 |
| Physical activity level (min/week), mean (SD) |  |  |  |
| Vigorous | 9.0 (40.3) | 16.0 (62.0) | 0.836 |
| Moderate | 36.0 (81.8) | 159.7 (393.2) | 0.179 |
| Light | 145.0 (186.5) | 164.7 (223.8) | 0.779 |
| Total | 190.0 (197.9) | 340.0 (384.4) | 0.141 |
| ^a^ *type of comorbidities*: musculoskeletal disease (15.0%), neurological disease (15.0%), endocrine disease (10.0%), gastrointestinal disease (15.0%), others oncological disease (10.0%), urological disease (10.0%).  ^b^ *type of comorbidities*: musculoskeletal disease (26.7%), neurological disease (20.0%), endocrine disease (20.0%), gastrointestinal disease (20.0%), others oncological disease (6.7%), cardiovascular disease (6.7%).  ^c^ one data missed due to multiple sclerosis in one patient | | | |

**Table S2.** Reasons for missed sessions of the entire cohort

| **Variable** | **Total cohort** | | |
| --- | --- | --- | --- |
|  | *Overall* | *Aerobic training* | *Resistance training* |
| Missed session, n (%) | 203 (100) | 105 (51.7) | 98 (48.3) |
| Reasons for the missed sessions |  |  |  |
| *Non-health related** | 60 (29.6) | 32 (30.5) | 28 (28.6) |
| *Oncological treatments scheduled on the same day* | 54 (26.6) | 28 (26.7) | 26 (26.5) |
| *Pain due to bone metastases* | 4 (2.0) | 0 (0.0) | 4 (4.1) |
| *Treatment-related side effects* | 23 (11.3) | 13 (12.4) | 10 (10.2) |
| *Fever* | 26 (12.8) | 14 (13.3) | 12 (12.2) |
| *Nephrostomy* | 6 (3.0) | 3 (2.9) | 3 (3.1) |
| *Vision problems due to cancer progression* | 20 (9.9) | 10 (9.5) | 10 (10.2) |
| *Follow-up visits* | 10 (4.9) | 5 (4.8) | 5 (5.1) |
| * Family constraints, vacation, personal reasons. | | | |

**Table S3.** Baseline patient characteristics by exercise program modality

| **Characteristics** | **Home-based program (n=12)** | **Gym-based program (n=8)** | **p-value** |
| --- | --- | --- | --- |
| Age, mean (SD) | 58.5 (8.4) | 64.3 (11.1) | 0.203 |
| Male, n (%) | 8 (33.3) | 4 (50.0) | 0.456 |
| Female, n (%) | 4 (66.7) | 4 (50.0) |  |
| Education, n (%) |  |  |  |
| Secondary | 1 (8.3) | 2 (25.0) | 0.402 |
| High school degree | 5 (41.7) | 2 (25.0) |  |
| Undergraduate degree | 4 (33.3) | 4 (50.0) |  |
| Postgraduate degree | 2 (16.7) | 0 (0.0) |  |
| Marital status, n (%) |  |  |  |
| Married | 9 (75.0) | 8 (100) | 0.308 |
| Divorced | 2 (16.7) | 0 (0.0) |  |
| Single | 1 (8.3) | 0 (0.0) |  |
| Employment, n (%) |  |  |  |
| Full-time employed | 6 (50.0) | 1(12.5) | 0.069 |
| Part-time employed | 0 (0.0) | 2 (25.0) |  |
| Retired | 4 (33.3) | 3 (37.5) |  |
| Sick leave | 2 (16.7) | 2 (25.0) |  |
| Family income, n (%) |  |  |  |
| More than adequate | 7 (83.3) | 3 (37.5) | 0.659 |
| Adequate | 4 (33.3) | 4 (50.0) |  |
| Barely Adequate | 1 (8.3) | 1 (12.5) |  |
| Stage, n (%) |  |  |  |
| III | 5 (41.7) | 2 (25.0) | 0.444 |
| IV | 7 (58.3) | 6 (75.0) |  |
| Tumor histology, n (%) |  |  |  |
| Adenocarcinoma | 10 (83.3) | 7 (87.5) | 0.416 |
| Neuroendocrine tumor | 0 (0.0) | 1 (12.5) |  |
| Acinar cell carcinoma | 1 (8.3) | 0 (0.0) |  |
| Colloid carcinoma | 1 (8.3) | 0 (0.0) |  |
| Tumor primary location, n (%) |  |  |  |
| Body of the pancreas | 5 (41.7) | 3 (37.5) | 0.783 |
| Head of pancreas | 4 (33.3) | 4 (50.0) |  |
| Body-tail of pancreas | 2 (16.7) | 1 (12.5) |  |
| Tail of pancreas | 1 (8.3) | 0 (0.0) |  |
| Metastatic involvement, n (%) |  |  |  |
| Single organ | 4 (57.1) | 4 (57.1) | 1.000 |
| Multiorgan | 3 (42.8) | 3 (42.8) |  |
| Metastases site, n (%) |  |  |  |
| Liver | 5 (41.7) | 4 (50.0) | 0.580 |
| Lung | 1 (8.3) | 4 (50.0) |  |
| Lymph nodes | 1 (8.3) | 2 (25.0) |  |
| Bone | 2 (16.7) | 1 (12.5) |  |
| Months since diagnosis, median (IQR) | 7.0 (4.0; 8.3) | 7.0 (4.3; 13.3) | 0.670 |
| Type of treatment, n (%) |  |  |  |
| Chemotherapy | 11 (83.3) | 8 (75.0) | 0.402 |
| Chemotherapy + immunotherapy | 1 (8.3) | 0 (0.0) |  |
| Prior surgery, n (%) |  |  |  |
| Yes | 4 (33.3) | 3 (37.5) | 0.848 |
| No | 8 (66.7) | 5 (62.5) |  |
| Comorbidities, n (%) |  |  |  |
| Diabetes | 2 (16.7) | 1(12.5) | 0.962 |
| Hypertension | 4 (33.3) | 2 (25.0) |  |
| Metabolic syndrome | 1 (8.3) | 1 (12.5) |  |
| Other | 13 (91.7)^a^ | 6 (75.0)^b^ |  |
| Anthropometric measures, mean (SD) |  |  |  |
| Body weight (kg) | 69.8 (10.6) | 66.2 (15.3) | 0.548 |
| Body mass index (kg/m^2^) | 23.8 (3.2) | 24.1 (5.3) | 0.879 |
| Waist (cm) | 85.6 (11.7) | 86.8 (17.0) | 0.844 |
| Hip (cm) | 96.6 (5.6) | 95.5 (7.7) | 0.721 |
| Waist-hip ratio (cm) | 0.9 (0.1) | 0.9 (0.1) | 0.688 |
| Chair sit and reach (cm), mean (SD) |  |  |  |
| Right leg | -2.8 (13.0) | -8.4 (12.1) | 0.342 |
| Left leg | -3.4 (13.5) | -10.8 (14.0) | 0.251 |
| Back scratch (cm), mean (SD) |  |  |  |
| Right arm | -7.5 (14.9) | -12.8 (15.1) | 0.446 |
| Left arm | -8.2 (13.6) | -8.6 (14.7) | 0.951 |
| Handgrip (kg) | 61.0 (21.2) | 57.3 (20.4) | 0.707 |
| 6-min walking test (m) | 575.8 (91.6)^c^ | 510.6 (114.6) | 0.186 |
| EORTC QLQ-C30, mean (SD) |  |  |  |
| Physical functioning | 86.1 (14.9) | 79.8 (10.6) | 0.208 |
| Role functioning | 81.9 (18.1) | 66.7 (12.6) | 0.050 |
| Emotional functioning | 68.8 (22.5) | 74.0 (19.1) | 0.407 |
| Cognitive functioning | 87.5 (14.4) | 82.3 (20.1) | 0.650 |
| Social functioning | 68.1 (19.4) | 68.8 (24.3) | 0.944 |
| Fatigue | 28.7 (18.6) | 45.1 (18.4) | 0.083 |
| Nausea/vomiting | 15.3 (16.6) | 16.7 (12.6) | 0.712 |
| Pain | 12.5 (19.0) | 22.9 (21.7) | 0.189 |
| Dyspnea | 19.4 (22.3) | 20.8 (17.2) | 0.794 |
| Insomnia | 20.0 (19.9) | 33.3 (17.8) | 0.017 |
| Appetite loss | 38.9 (39.8) | 33.3 (30.9) | 0.839 |
| Constipation | 5.6 (13.0) | 8.3 (15.4) | 0.697 |
| Diarrhea | 19.4 (22.3) | 15.7 (30.9) | 0.567 |
| Financial problems | 8.3 (15.1) | 16.7 (17.8) | 0.283 |
| Global health status | 61.1 (21.1) | 55.2 (16.6) | 0.390 |
| Physical activity level (min/week) , mean (SD) |  |  |  |
| Vigorous | 15.0 (52.0) | 0.0 (0.0) | 0.475 |
| Moderate | 37.5 (75.8) | 33.8 (95.5) | 0.659 |
| Light | 168.3 (233.7) | 110.0 (79.2) | 0.690 |
| Total | 220.8 (240.2) | 143.8 (107.4) | 0.814 |
| ^a^ *type of comorbidities*: musculoskeletal disease (8.3%), neurological disease (25.0%), endocrine disease (16.7%), gastrointestinal disease (8.3%), others oncological disease (8.3%), urological disease (25.0%),  ^b^ *type of comorbidities*: musculoskeletal disease (25.0%), neurological disease (12.5%), gastrointestinal disease (25.0%), others oncological disease (12.5%).  ^c^ one data missed due to multiple sclerosis in one patient | | | |

**Table S4.** Feasibility outcomes by exercise program modality

| **Variable** | **Home-based program** | | | **Gym-based program** | | |
| --- | --- | --- | --- | --- | --- | --- |
|  | *Overall* | *Aerobic training* | *Resistance*  *training* | *Overall* | *Aerobic training* | *Resistance*  *training* |
| Lost to follow-up, n (%) | 11 (73) | 0 (0) | 0 (0) | 4 (27) | 0 (0) | 0 (0) |
| Adherence, median (IQR) | 79% (72%-94%) | 81% (71%-94%) | 81% (71%-100%) | 73% (64%-81%) | 77% (70%-81%) | 75% (64%-81%) |
| Attendance, median (IQR) | 79% (72%-94%) | 81% (71%-94%) | 81% (71%-100%) | 77% (70%-84%) | 79% (70%-84%) | 79% (70%-84%) |
| Treatment interruption, n (%) | 4 (33) | - | - | 4 (50) | - | - |
| Permanent discontinuation, n (%) | 2 (17) | - | - | 0 (0) | - | - |
| Missed session, n (%) | 115 (57) | 61 (53) | 54 (47) | 88 (43) | 44 (50) | 44 (50) |
| Dose modification, n (%) | 1 (8) | - | - | 1 (13) | - | - |
| Early session termination, n (%) | 0 (0) | 0 (0) | 0 (0) | 0 (0) | 0 (0) | 0 (0) |
| Tolerability, % | 100 | 100 | 100 | 100 | 100 | 100 |
| Definition: ***Lost to follow-up***, number of patients who did not complete the study; ***Adherence***, number of completed planned exercise dosage compared to the total programmed; ***Attendance***, number of attended sessions compared to the total prescribed; ***Treatment interruption***, number of patients who missed ≥3 continuous sessions; ***Permanent discontinuation***, number of patients who ended the program before concluding the 12 weeks; ***Missed session***, number of sessions not attended by the patients; ***Dose modification***, number of patients that required ≥10% of sessions dose escalation/reduction; ***Early session termination***, number of sessions interrupted before the planned intensity/duration; ***Tolerability***, number sessions performed at the planned intensity. | | | | | | |

| **Variable** | **Home-based program** | | | **Gym-based program** | | |
| --- | --- | --- | --- | --- | --- | --- |
|  | *Overall* | *Aerobic training* | *Resistance*  *training* | *Overall* | *Aerobic training* | *Resistance*  *training* |
| Missed session, n (%) | 115 (100) | 61 (53.0) | 54 (47.0) | 88 (100) | 44 (50.0) | 44 (50.0) |
| Reasons for the missed sessions |  |  |  |  |  |  |
| *Non-health related** | 32 (27.8) | 18 (29.5) | 14 (25.9) | 28 (31.8) | 14 (31.8) | 14 (31.8) |
| *Oncological treatments scheduled on the same day* | 38 (33.0) | 20 (32.8) | 18 (33.3) | 16 (18.2) | 8 (18.2) | 8 (18.2) |
| *Pain due to bone metastases* | 4 (3.5) | 0 (0.0) | 4 (7.4) | 0 (0.0) | 0 (0.0) | 0 (0.0) |
| *Treatment-related side effects* | 5 (4.3) | 4 (6.6) | 1 (1.9) | 18 (20.5) | 9 (20.5) | 9 (20.5) |
| *Fever* | 16 (13.9) | 9 (14.8) | 7 (13.0) | 10 (11.4) | 5 (11.4) | 5 (11.4) |
| *Nephrostomy* | 0 (0.0) | 0 (0.0) | 0 (0.0) | 6 (6.8) | 3 (6.8) | 3 (6.8) |
| *Vision problems due to cancer progression* | 20 (17.4) | 10 (16.4) | 10 (18.5) | 0 (0.0) | 0 (0.0) | 0 (0.0) |
| *Follow-up visits* | 0 (0.0) | 0 (0.0) | 0 (0.0) | 10 (11.4) | 5 (11.4) | 5 (11.4) |
| * Family constraints, vacation, personal reasons. | | | | | | |

**Table S5.** Reasons for missed sessions by exercise program modality

**Table S6.** Changes in physical fitness by exercise program modality

| Variable | Baseline, mean (SD) | Postintervention, mean (SD) | p-value  (in-group) | p-value  (between-group) |
| --- | --- | --- | --- | --- |
| Anthropometric measures |  |  |  |  |
| Body weight (kg) |  |  |  |  |
| Home-based program | 69.83 (10.61) | 71.46 (11.18) | 0.087 | 0.246 |
| Gym-based program | 66.29 (15.35) | 66.26 (14.31) | 0.978 |  |
| Body mass index (kg/m^2^) |  |  |  |  |
| Home-based program | 23.84 (3.15) | 24.47 (3.40) | 0.080 | 0.203 |
| Gym-based program | 24.13 (5.33) | 24.08 (4.54) | 0.909 |  |
| Waist (cm) |  |  |  |  |
| Home-based program | 85.60 (11.71) | 86.79 (12.66) | 0.455 | 0.070 |
| Gym-based program | 86.88 (17.01) | 83.64 (14.13) | 0.082 |  |
| Hip (cm) |  |  |  |  |
| Home-based program | 96.58 (5.64) | 99.03 (5.98) | 0.049 | 0.026 |
| Gym-based program | 95.50 (7.73) | 92.49 (5.23) | 0.216 |  |
| Waist-hip ratio (cm) |  |  |  |  |
| Home-based program | 0.89 (0.10) | 0.87 (0.09) | 0.319 | 0.535 |
| Gym-based program | 0.90 (0.12) | 0.90 (0.15) | 0.972 |  |
| Chair sit and reach (cm) |  |  |  |  |
| Right leg |  |  |  |  |
| Home-based program | -2.79 (13.04) | -1.71 (14.33) | 0.632 | 0.606 |
| Gym-based program | -8.44 (12.07) | -9.25 (14.53) | 0.791 |  |
| Left leg |  |  |  |  |
| Home-based program | -3.42 (13.46) | -3.54 (14.87) | 0.946 | 1.000 |
| Gym-based program* | -12.75 (-23.50; -0.25) | -9.25 (-18.50; -2.75) | 0.742 |  |
| Back scratch (cm) |  |  |  |  |
| Right arm |  |  |  |  |
| Home-based program | -7.48 (14.90) | -4.67 (13.20) | 0.06 | 0.314 |
| Gym-based program | -12.81 (15.12) | -12.38 (14.85) | 0.832 |  |
| Left arm |  |  |  |  |
| Home-based program | -8.17 (13.61) | -6.87 (13.80) | 0.296 | 0.453 |
| Gym-based program* | -4.75 (-21.50; 4.75) | -4.75 (-20.50; 2.00) | 0.688 |  |
| Handgrip (kg) |  |  |  |  |
| Home-based program | 60.96 (21.20) | 64.37 (17.77) | 0.328 | 0.908 |
| Gym-based program* | 49.50 (44.50; 72.75) | 49.00 (45.75; 62.50) | 0.742 |  |
| 6-min walking test (m) |  |  |  |  |
| Home-based program | 569.40 (93.93) | 576.64 (57.42) | 0.685 | 0.152 |
| Gym-based program | 510.61 (114.64) | 553.20 (92.29) | 0.052 |  |
| ^*^ Data presented as median and interquartile range | | | | |

**Table S7**. Changes in quality of life and physical activity levels by exercise program modality

| Variable | Baseline, median (IQR) | Postintervention, median (IQR) | p-value  (in-group) | p-value  (between-group) |
| --- | --- | --- | --- | --- |
| EORTC QLQ C-30 |  |  |  |  |
| Physical functioning |  |  |  |  |
| Home-based program* | 86.11 (14.90) | 87.78 (17.25) | 0.555 | 0.584 |
| Gym-based program | 80.00 (76.67; 86.67) | 86.67 (80.00; 86.67) | 0.375 |  |
| Role functioning |  |  |  |  |
| Home-based program* | 81.94 (18.06) | 93.06 (19.41) | 0.087 | 0.605 |
| Gym-based program * | 66.67 (12.60) | 72.92 (23.47) | 0.402 |  |
| Emotional functioning |  |  |  |  |
| Home-based program | 75.00 (62.50; 79.17) | 83.33 (75.00; 95.83) | 0.078 | 1.000 |
| Gym-based program* | 73.96 (19.13) | 82.29 (8.26) | 0.155 |  |
| Cognitive functioning |  |  |  |  |
| Home-based program | 91.67 (75.00; 100.00) | 100.000 (83.33; 100.00) | 0.58 | 0.715 |
| Gym-based program | 83.33 (75.00; 100.00) | 91.67 (83.33; 100.00) | 0.250 |  |
| Social functioning |  |  |  |  |
| Home-based program | 66.67 (58.33; 75.00) | 83.33 (75.00; 100.00) | 0.023 | 0.158 |
| Gym-based program* | 68.75 (24.30) | 68.75 (18.77) | 1.000 |  |
| Fatigue |  |  |  |  |
| Home-based program* | 28.70 (18.63) | 19.44 (19.03) | 0.147 | 0.754 |
| Gym-based program* | 45.14 (18.41) | 37.50 (15.64) | 0.323 |  |
| Nausea/vomiting |  |  |  |  |
| Home-based program | 16.67 (0.00; 25.00) | 8.33 (0.00; 33.33) | 0.688 | 1.000 |
| Gym-based program | 16.67 (8.33; 25.00) | 8.33 (0.00; 25.00) | 0.875 |  |
| Pain |  |  |  |  |
| Home-based program | 0.00 (0.00; 16.67) | 0.00 (0.00; 0.00) | 0.125 | 0.083 |
| Gym-based program* | 22.92 (21.71) | 27.08 (25.10) | 0.598 |  |
| Dyspnea |  |  |  |  |
| Home-based program | 16.67 (0.00; 33.33) | 0.00 (0.00; 33.33) | 0.438 | 0.539 |
| Gym-based program | 33.33 (0.00; 33.33) | 16.67 (0.00; 33.33) | 1.000 |  |
| Insomnia |  |  |  |  |
| Home-based program | 0.00 (0.00; 33.33) | 0.00 (0.00; 33.33) | 1.000 | 0.381 |
| Gym-based program* | 33.33 (17.82) | 20.83 (30.54) | 0.351 |  |
| Appetite loss |  |  |  |  |
| Home-based program | 33.00 (0.00; 66.67) | 0.00 (0.00; 16.67) | 0.031 | 0.144 |
| Gym-based program* | 33.33 (30.86) | 25.00 (29.56) | 0.351 |  |
| Constipation |  |  |  |  |
| Home-based program | 0.00 (0.00; 0.00) | 33.33 (0.00; 33.33) | 0.031 | 0.052 |
| Gym-based program | 0.00 (0.00; 8.33) | 0.00 (0.00; 0.00) | 1.000 |  |
| Diarrhea |  |  |  |  |
| Home-based program | 16.67 (0.00; 33.33) | 0.00 (0.00; 16.67) | 0.125 | 0.048 |
| Gym-based program | 0.00 (0.00; 66.67) | 0.00 (0.00; 75.00) | 1.000 |  |
| Financial problems |  |  |  |  |
| Home-based program | 0.00 (0.00; 16.67) | 0.00 (0.00; 0.00) | 0.500 | 0.697 |
| Gym-based program | 16.67 (0.00; 33.33) | 0.00 (0.00; 16.67) | 0.500 |  |
| Global health status |  |  |  |  |
| Home-based program* | 61.11 (21.12) | 72.92 (19.17) | 0.027 | 0.039 |
| Gym-based program* | 55.21 (16.63) | 50.00 (9.96) | 0.435 |  |
| Physical activity level (min/week) |  |  |  |  |
| Vigorous |  |  |  |  |
| Home-based program | 0.00 (0.00; 0.00) | 0.00 (0.00; 90.00) | 0.250 | 0.154 |
| Gym-based program | 0.00 (0.00; 0.00) | 0.00 (0.00; 0.00) | 1.000 |  |
| Moderate |  |  |  |  |
| Home-based program | 0.00 (0.00; 45.00) | 157.50 (90.00; 360.00) | <0.001 | 0.044 |
| Gym-based program | 0.00 (0.00; 0.00) | 30.00 (0.00; 90.00) | 0.125 |  |
| Light |  |  |  |  |
| Home-based program | 0.00 (0.00; 320.00) | 27.50 (0.00; 105.00) | 0.625 | 0.197 |
| Gym-based program* | 110.00 (79.28) | 152.50 (134.88) | 0.444 |  |
| Total |  |  |  |  |
| Home-based program | 165.00 (0.00; 440.00) | 390.00 (157.5; 555.00) | 0.064 | 0.177 |
| Gym-based program* | 143.75 (107.43) | 227.50 (149.36) | 0.104 |  |
| ^*^ Data presented as mean and standard deviation | | | | |

**Table S8.** Baseline patient characteristics by tumor stage

| **Characteristics** | **Stage III (n=7)** | **Stage IV (n=13)** | **p-value** |
| --- | --- | --- | --- |
| Age, mean (SD) | 62.0 (8.3) | 60.1 (10.7) | 0.689 |
| Male, n (%) | 6 (85.7) | 6 (46.2) | 0.085 |
| Female, n (%) | 1 (14.3) | 7 (53.8) |  |
| Education, n (%) |  |  |  |
| Secondary | 1 (14.3) | 2 (15.4) | 0.074 |
| High school degree | 5 (71.4) | 2 (15.4) |  |
| Undergraduate degree | 1(14.2) | 7 (53.8) |  |
| Postgraduate degree | 0 (0.0) | 2 (15.4) |  |
| Marital status, n (%) |  |  |  |
| Married | 5 (71.4) | 12 (92.3) | 0.106 |
| Divorced | 2 (28.6) | 0 (0.0) |  |
| Single | 0 (0.0) | 1 (7.7) |  |
| Employment, n (%) |  |  |  |
| Full-time employed | 3 (42.9) | 4 (30.7) | 0.483 |
| Part-time employed | 0 (0.0) | 2 (15.38) |  |
| Retired | 2 (28.6) | 5 (38.5) |  |
| Sick leave | 2 (28.6) | 2 (15.4) |  |
| Family income, n (%) |  |  |  |
| More than adequate | 10 (50.0) | 7 (53.8) | 0.848 |
| Adequate | 8 (40.0) | 5 (38.5) |  |
| Barely Adequate | 2 (10.0) | 1 (7.7) |  |
| Tumor histology, n (%) |  |  |  |
| Adenocarcinoma | 5 (71.4) | 12 (92.3) | 0.214 |
| Neuroendocrine tumor | 1 (14.3) | 0 (0.0) |  |
| Acinar cell carcinoma | 0 (0.0) | 1 (7.7) |  |
| Colloid carcinoma | 1 (14.3) | 0 (0.0) |  |
| Tumor primary location, n(%) |  |  |  |
| Body of the pancreas | 2 (28.6) | 6 (46.2) | 0.525 |
| Head of the pancreas | 3 (42.9) | 5 (38.5) |  |
| Body-tail of the pancreas | 1 (14.3) | 2 (15.4) |  |
| Tail of the pancreas | 1 (14.3) | 0 (0.0) |  |
| Months since diagnosis, median (IQR) | 8.0 (5.0; 9.5) | 6.0 (4.0; 8.0) | 0.710 |
| Type of treatment, n (%) |  |  |  |
| Chemotherapy | 7 (100) | 12 (92.3) | 0.451 |
| Chemotherapy + immunotherapy | 0 (0.0) | 1 (7.7) |  |
| Prior surgery, n (%) |  |  |  |
| Yes | 3 (42.9) | 4 (30.8) | 0.589 |
| No | 4 (57.1) | 9 (69.2) |  |
| Comorbidities, n (%) |  |  |  |
| Diabetes | 1 (14.3) | 2 (15.4) | 0.743 |
| Hypertension | 3 (42.9) | 3(23.1) |  |
| Metabolic syndrome | 1 (14.3) | 1 (7.7) |  |
| Other | 4 (57.1)^a^ | 11 (84.6)^b^ |  |
| Exercise program methods, n (%) |  |  |  |
| Gym-based program | 2 (28.7) | 6 (46.1) | 0.444 |
| Home-based program | 5 (71.4) | 7 (53.8) |  |
| Anthropometric measures, mean (SD) |  |  |  |
| Body weight (kg) | 72.6 (5.3) | 66.2 (14.7) | 0.285 |
| Body mass index (kg/m^2^) | 24.9 (1.9) | 23.4 (4.8) | 0.452 |
| Waist (cm) | 90.3 (5.8) | 83.8 (16.2) | 0.325 |
| Hip (cm) | 97.9 (4.7) | 95.2 (7.1) | 0.375 |
| Waist-hip ratio (cm) | 0.9 (0.1) | 0.9 (0.1) | 0.322 |
| Chair sit and reach (cm), mean (SD) |  |  |  |
| Right leg | -5.6 (12.6) | -5.0 (13.8) | 0.915 |
| Left leg | -9.2 (9.7) | -4.6 (15.17) | 0.480 |
| Back scratch (cm), mean (SD) |  |  |  |
| Right arm | -9.8 (12.7) | -5.2 (14.9) | 0.502 |
| Left arm | -12.9 (11.7) | -10.2 (15.7) | 0.704 |
| Handgrip (kg) | 64.6 (24.4) | 56.8 (18.4) | 0.430 |
| 6-min walking test (m) | 575.2 (85.0)^c^ | 536.0 (113.0) | 0.462 |
| EORTC QLQ-C30, mean (SD) |  |  |  |
| Physical functioning | 77.9 (17.0) | 86.7 (10.5) | 0.167 |
| Role functioning | 69.0 (20.2) | 79.5 (15.4) | 0.212 |
| Emotional functioning | 64.3 (31.1) | 74.4 (12.9) | 0.316 |
| Cognitive functioning | 84.5 (22.8) | 85.9 (13.3) | 0.799 |
| Social functioning | 66.7 (16.7) | 69.2 (23.4) | 0.801 |
| Fatigue | 37.3 (24.6) | 34.2 (17.8) | 0.748 |
| Nausea/vomiting | 16.7 (16.7) | 15.4 (14.4) | 0.866 |
| Pain | 14.3 (17.8) | 17.9 (22.0) | 0.866 |
| Dyspnea | 28.6 (23.0) | 15.4 (17.3) | 0.211 |
| Insomnia | 19.0 (26.2) | 20.5 (16.8) | 0.721 |
| Appetite loss | 28.6 (40.5) | 41.0 (33.8) | 0.428 |
| Constipation | 14.3 (17.8) | 2.6 (9.2) | 0.077 |
| Diarrhea | 14.3 (17.8) | 20.5 (29.0) | 0.856 |
| Financial problems | 14.3 (17.8) | 10.3 (16.0) | 0.632 |
| Global health status | 53.8 (18.5) | 61.5 (19.7) | 0.391 |
| Physical activity level (min/week), mean (SD) |  |  |  |
| Vigorous | 0.0 (0.0) | 13.8 (49.9) | 0.529 |
| Moderate | 17.1 (45.4) | 46.2 (96.1) | 0.650 |
| Light | 55.7 (95.5) | 193.1 (208.2) | 0.065 |
| Total | 72.9 (94.6) | 253.1 (212.6) | 0.044 |
| ^a^ *type of comorbidities*: neurological disease (14.3%), endocrine disease (14.3%), gastrointestinal disease (14.3%), urological disease (14.3%).  ^b^ *type of comorbidities*: musculoskeletal disease (23.1%), neurological disease (23.1%), endocrine disease (7.7%), gastrointestinal disease (15.4%), others oncological disease (15.3%), urological disease (15.4%).  ^c^ one data missed due to multiple sclerosis in one patient | | | |

**Table S9.** Feasibility outcomes by tumor stage

| **Variable** | **Stage III** | | | **Stage IV** | | |
| --- | --- | --- | --- | --- | --- | --- |
|  | *Overall* | *Aerobic training* | *Resistance*  *training* | *Overall* | *Aerobic training* | *Resistance*  *training* |
| Lost to follow-up, n (%) | 2 (13) | 0 (0) | 0 (0) | 13 (87) | 0 (0) | 0 (0) |
| Adherence, median (IQR) | 79% (83%; 62%) | 79% (83%; 71%) | 79% (83%; 60%) | 78% (96%; 74%) | 83% (96%; 74%) | 83% (96%; 74%) |
| Attendance, median (IQR) | 79% (85%; 73%) | 79% (85%; 73%) | 79% (85%; 73%) | 75% (92%; 71%) | 79% (92%; 71%) | 79% (92%; 71%) |
| Treatment interruption, n (%) | 4 (33) | 0 (0) | 0 (0) | 4 (50) | 0 (0) | 0 (0) |
| Permanent discontinuation, n (%) | 1 (14) | 0 (0) | 0 (0) | 1 (8) | 0 (0) | 0 (0) |
| Missed session, n (%) | 72 (35) | 36 (50) | 36 (50) | 131 (65) | 69 (53) | 62 (47) |
| Dose modification, n (%) | 1 (50) | 0 (0) | 0 (0) | 1 (50) | 0 (0) | 0 (0) |
| Early session termination, n (%) | 0 (0) | 0 (0) | 0 (0) | 0 (0) | 0 (0) | 0 (0) |
| Tolerability, % | 100 | 100 | 100 | 100 | 100 | 100 |
| Definition: ***Lost to follow-up***, number of patients who did not complete the study; ***Adherence***, number of completed planned exercise dosage compared to the total programmed; ***Attendance***, number of attended sessions compared to the total prescribed; ***Treatment interruption***, number of patients who missed ≥3 continuous sessions; ***Permanent discontinuation***, number of patients who ended the program before concluding the 12 weeks; ***Missed session***, number of sessions not attended by the patients; ***Dose modification***, number of patients that required ≥10% of sessions dose escalation/reduction; ***Early session termination***, number of sessions interrupted before the planned intensity/duration; ***Tolerability***, number sessions performed at the planned intensity. | | | | | | |

**Table S10.** Reasons for missed sessions by tumor stage

| **Variable** | **Total cohort** | | | **Stage III** | | | **Stage IV** | | |
| --- | --- | --- | --- | --- | --- | --- | --- | --- | --- |
|  | *Overall* | *Aerobic training* | *Resistance*  *training* | *Overall* | *Aerobic training* | *Resistance*  *training* | *Overall* | *Aerobic training* | *Resistance*  *training* |
| Missed session, n (%) | 203 (100) | 105 (51.7) | 98 (48.3) | 72 (100) | 36 (50.0) | 36 (50.0) | 131 (100) | 69 (52.7) | 62 (47.3) |
| Reasons for the missed sessions |  |  |  |  |  |  |  |  |  |
| *Non-health related** | 60 (29.6) | 32 (30.5) | 28 (28.6) | 22 (30.6) | 11 (30.6) | 11 (30.6) | 38 (29) | 21 (30.4) | 17 (27.4) |
| *Oncological treatments scheduled on the same day* | 54 (26.6) | 28 (26.7) | 26 (26.5) | 30 (41.7) | 15 (41.7) | 15 (41.7) | 24 (18.3) | 13 (18.8) | 11 (17.7) |
| *Pain due to bone metastases* | 4 (2.0) | 0 (0) | 4 (4.1) | 0 (0) | 0 (0) | 0 (0) | 4 (3.1) | 0 (0) | 4 (6.5) |
| *Treatment-related side effects* | 23 (11.3) | 13 (12.4) | 10 (10.2) | 2 (2.8) | 1 (2.8) | 1 (2.8) | 21 (16.0) | 12 (17.4) | 9 (14.5) |
| *Fever* | 26 (12.8) | 14 (13.3) | 12 (12.2) | 12 (16.7) | 6 (16.7) | 6 (16.7) | 14 (10.7) | 8 (11.6) | 6 (9.7) |
| *Nephrostomy* | 6 (3) | 3 (2.9) | 3 (3.1) | 0 (0) | 0 (0) | 0 (0) | 6 (4.6) | 3 (4.3) | 3 (4.8) |
| *Vision problems due to cancer progression* | 20 (9.9) | 10 (9.5) | 10 (10.2) | 0 (0) | 0 (0) | 0 (0) | 20 (15.3) | 10 (14.5) | 10 (16.1) |
| *Follow-up visits* | 10 (4.9) | 5 (4.8) | 5 (5.1) | 6 (8.3) | 3 (8.3) | 3 (8.3) | 4 (3.1) | 2(2.9) | 2 (3.2) |
| * Family constraints, vacation, personal reasons. | | | | | | | | | |

**Table S11.** Changes in physical fitness by tumor stage

| Variable | Baseline, mean (SD) | Postintervention, mean (SD) | p-value  (in-group) | p-value  (between-group) |
| --- | --- | --- | --- | --- |
| Anthropometric measures |  |  |  |  |
| Body weight (kg) |  |  |  |  |
| Stage III | 72.57 (5.30) | 73.75 (5.49) | 0.283 | 0.829 |
| Stage IV | 66.17 (14.70) | 67.03 (14.58) | 0.380 |  |
| Body mass index (kg/m^2^) |  |  |  |  |
| Stage III | 24.91(1.89) | 25.35 (2.03) | 0.250 | 0.648 |
| Stage IV | 23.44 (4.81) | 23.68 (4.39) | 0.470 |  |
| Waist (cm) |  |  |  |  |
| Stage III | 90.31 (5.78) | 89.43 (7.25) | 0.718 | 0.858 |
| Stage IV | 83.85 (16.20) | 83.43 (15.09) | 0.776 |  |
| Hip (cm) |  |  |  |  |
| Stage III | 97.93 (4.70) | 98.43 (6.83) | 0.827 | 0.891 |
| Stage IV | 95.19 (7.11) | 95.32 (6.23) | 0.934 |  |
| Waist-hip ratio (cm) |  |  |  |  |
| Stage III | 0.92 (0.06) | 0.91 (0.07) | 0.566 | 0.582 |
| Stage IV | 0.88 (0.12) | 0.87 (0.13) | 0.840 |  |
| Chair sit and reach (cm) |  |  |  |  |
| Right leg |  |  |  |  |
| Stage III | -5.64 (12.64) | -9.79 (10.07) | 0.161 | 0.173 |
| Stage IV | -4.96 (13.81) | -2.62 (5.82) | 0.355 |  |
| Left leg |  |  |  |  |
| Stage III | -9.21 (9.67) | -9.79 (10.07) | 0.762 | 0.751 |
| Stage IV* | 0.00 (-18.13; 9.50) | 2.00 (-20.50; 9.75) | 1.000 |  |
| Back scratch (cm) |  |  |  |  |
| Right arm |  |  |  |  |
| Stage III | -9.76 (12.70) | -6.93 (12.38) | 0.206 | 0.874 |
| Stage IV | -5.19 (14.91) | -4.08 (13.31) | 0.413 |  |
| Left arm |  |  |  |  |
| Stage III | -12.86 (11.70) | -11.79 (12.60) | 0.556 | 0.468 |
| Stage IV* | -4.00 (-23.25; 2.13) | -6.00 (-19.5; 3.25) | 0.365 |  |
| Handgrip (kg) |  |  |  |  |
| Stage III | 64.57 (24.44) | 72.86 (16.82) | 0.054 | 0.163 |
| Stage IV | 56.77 (18.42) | 58.96 (17.10) | 0.039 |  |
| 6-min walking test (m) |  |  |  |  |
| Stage III* | 559.25 (522.60; 667.00) | 581.70 (568.00; 620.00) | 1.000 | 0.246 |
| Stage IV | 527.31 (113.39) | 560.69 (84.90) | 0.031 |  |
| ^*^Data presented as median and interquartile range | | | | |

**Table S12.** Changes in quality of life and physical activity levels by tumor stage

| Variable | Baseline, median (IQR) | Postintervention, median (IQR) | p-value  (in-group) | p-value  (between-group) |
| --- | --- | --- | --- | --- |
| EORTC QLQ C-30 |  |  |  |  |
| Physical functioning |  |  |  |  |
| Stage III* | 77.86 (16.96) | 84.76 (21.33) | 0.233 | 0.188 |
| Stage IV | 86.67 (80.00; 95.00) | 86.67 (80.00; 93.00) | 0.910 |  |
| Role functioning |  |  |  |  |
| Stage III | 66.67 (50.00; 83.33) | 100.00 (83.33; 100.00) | 0.031 | 0.230 |
| Stage IV | 83.33 (66.67; 87.50) | 100.00 (62.5; 100.00) | 0.938 |  |
| Emotional functioning |  |  |  |  |
| Stage III* | 64.29 (31.07) | 90.48 (12.20) | 0.072 | 0.041 |
| Stage IV* | 74.36 (12.94) | 77.56 (14.98) | 0.037 |  |
| Cognitive functioning |  |  |  |  |
| Stage III | 100.00 (70.83; 100.00) | 100.00 (87.50; 100.00) | 0.250 | 0.072 |
| Stage IV | 83.33 (79.17; 100.00) | 100.00 (83.33; 100.00) | 0.641 |  |
| Social functioning |  |  |  |  |
| Stage III* | 66.67 (16.67) | 78.57 (24.93) | 0.334 | 0.646 |
| Stage IV | 66.67 (50.00; 87.50) | 83.33 (66.67; 87.50) | 0.129 |  |
| Fatigue |  |  |  |  |
| Stage III | 33.33 (33.33; 41.67) | 33.33 (2.78; 33.33) | 0.250 | 0.806 |
| Stage IV* | 34.19 (17.84) | 29.06 (20.56) | 0.323 |  |
| Nausea/vomiting |  |  |  |  |
| Stage III* | 16.67 (16.67) | 0.00 (0.00) | 0.038 | 0.300 |
| Stage IV | 16.67 (0.00; 16.67) | 16.67 (12.50; 33.33) | 0.219 |  |
| Pain |  |  |  |  |
| Stage III | 16.67 (0.00; 16.67) | 0.00 (0.00; 0.00) | 0.125 | 0.010 |
| Stage IV | 16.67 (0.00; 33.33) | 16.67 (0.00; 33.33) | 1.000 |  |
| Dyspnea |  |  |  |  |
| Stage III | 33.33 (8.33; 33.33) | 0.00 (0.00; 25.00) | 0.250 | 0.057 |
| Stage IV | 0.00 (0.00; 33.33) | 0.00 (0.00; 33.33) | 1.000 |  |
| Insomnia |  |  |  |  |
| Stage III | 0.00 (0.00; 33.33) | 0.00 (0.00; 0.00) | 0.500 | 0.087 |
| Stage IV* | 20.51 (16.88) | 20.51 (25.60) | 1.000 |  |
| Appetite loss |  |  |  |  |
| Stage III* | 28.57 (40.50) | 9.52 (16.27) | 0.280 | 0.368 |
| Stage IV | 33.00 (0.00; 66.67) | 0.00 (0.00; 33.33) | 0.031 |  |
| Constipation |  |  |  |  |
| Stage III* | 14.29 (17.82) | 23.81 (25.20) | 0.356 | 0.701 |
| Stage IV | 0.00 (0.00; 0.00) | 0.00 (0.00; 33.33) | 0.125 |  |
| Diarrhea |  |  |  |  |
| Stage III | 0.00 (0.00; 33.33) | 0.00 (0.00; 0.00) | 0.500 | 0.820 |
| Stage IV | 0.00 (0.00; 41.67) | 0.00 (0.00; 33.33) | 1.000 |  |
| Financial problems |  |  |  |  |
| Stage III | 0.00 (0.00; 33.33) | 0.00 (0.00; 0.00) | 0.500 | 0.434 |
| Stage IV | 0.00 (0.00; 33.33) | 0.00 (0.00; 0.00) | 0.500 |  |
| Global health status |  |  |  |  |
| Stage III* | 53.57 (18.55) | 75.00 (14.43) | 0.012 | 0.530 |
| Stage IV* | 61.54 (19.70) | 57.69 (19.68) | 0.307 |  |
| Physical activity level (min/week) |  |  |  |  |
| Vigorous |  |  |  |  |
| Stage III | 0.00 (0.00; 00.00) | 0.00 (0.00; 90.00) | 0.500 | 0.397 |
| Stage IV | 0.00 (0.00; 0.00) | 0.00 (0.00; 0.00) | 1.000 |  |
| Moderate |  |  |  |  |
| Stage III* | 17.14 (45.36) | 138.57 (118.38) | 0.037 | 0.202 |
| Stage IV | 0.00 (0.00; 22.50) | 90.00 (0.00; 360.00) | 0.004 |  |
| Light |  |  |  |  |
| Stage III* | 55.71 (95.54) | 105.00 (122.78) | 0.485 | 0.749 |
| Stage IV | 90.00 (45.00; 300.00) | 60.00 (32.5; 123.75) | 0.765 |  |
| Total |  |  |  |  |
| Stage III* | 72.86 (94.64) | 286.43 (172.31) | 0.054 | 0.474 |
| Stage IV* | 253.07 (212.62) | 350.42 (231.52) | 0.232 |  |
| ^*^ Data presented as mean and standard deviation | | | | |
